# Job Strain Does Not Relate to Morning Level Vanilmandelic Acid in Japanese Civil Servants

**DOI:** 10.2188/jea.12.324

**Published:** 2007-11-30

**Authors:** Ali Nasermoaddeli, Michikazu Sekine, Shimako Hamanishi, Sadanobu Kagamimori

**Affiliations:** Department of Welfare Promotion and Epidemiology, Toyama Medical and Pharmaceutical University.

**Keywords:** job strain, vanilmandelic acid, sympathoadrenal activity, smoking, civil servants

## Abstract

Epidemiologic and clinical studies have related acute and, less frequently, chronic life stress to cardiovascular diseases. In addition, animal models suggest that chronic psychological stress could cause atherosclerosis, probably by increasing sympathetic activation. In this cross-sectional study we evaluated the association between job strain, as one of the markers of workplace stress, and the urinary excretion of Vanilmandelic acid (VMA) upon awakening as a sympathoadrenal activity marker in the morning. Subjects were 936 male and 823 female civil servants working in departments related to the municipality of a city in Toyama Prefecture, Japan, in the spring of 2001. VMA was measured by high-performance liquid chromatography. We found that there was an age dependent increase in the level of VMA and females had higher VMA levels than males. Males who were current smokers had significantly lower VMA levels than nonsmokers after adjusting for age. Job strain level did not relate to VMA concentration in urine after adjusting for age and smoking status both in men and women. In addition, working and sleeping hours as predictor variables were also not associated with urinary VMA levels upon awakening in the morning. In conclusion, it seems that job strain does not independently relate to the sympathoadrenal activity, but the interaction between job strain and other variables such as personal characteristics and environmental factors and their relation with sympathoadrenal activity should further be explored.

Anecdotal reports and case studies^1^^-^^3^^)^ have long reported a relationship between acute life stress and the development of cardiac diseases. The effects of acute stress on heart disease are well supported by epidemiologic studies investigating natural life stressors.^4^^-^^5^ In contrast to chronic stress, however, acute stress is easier to model and can be studied under controlled laboratory conditions in both humans and animals.

Work related stress is the most widely studied chronic life stress in relation to coronary artery diseases (CAD).^6^^-^^10^ Although many aspects of one’s work environment have been studied, much interest has focused on models of inherent tension at work and its relation to the development of CAD. One such model is the ‘job strain’ model proposed by Karasek,^11^^,^^12^ where job strain is defined as jobs with high demand but low decision latitude (control). Although the studies regarding presence of stress at work and subsequent CAD development have been largely positive^6^^-^^9^^,^^13^ suggesting a causal relationship between this form of chronic stress and development of CAD, the pathophysiologic pathway through which work related stress causes CAD has not been fully addressed.

An extensive body of evidence from animal models revealed that chronic psychological stress could lead, probably via a mechanism involving excessive sympathetic nervous system activation, to exacerbation of coronary artery atherosclerosis as well as to transient endothelial dysfunction and even necrosis.^14^ However, little is known about the long lasting effects of psychosocial stress at work on sympathoadrenal activity, which might be a pathologic burden for CAD incidence in humans.^15^^-^^17^ Thus, this study was conducted to evaluate the impact of the job strain model and its dimensions (job demand and control) on vanilmandelic acid (VMA) as a metabolite to mark sympathoadrenal activity among Japanese civil servants. An increase in excretion of VMA upon awakening in the morning was expected with increasing level of job strain. According to previous reports,^18^^,^^19^ VMA excretion is less changed by various quantities of foods, in addition, due to the number of our study population we decided to measure one sympathoadrenal marker rather than assessing adrenaline and noradrenaline together.

## METHODS

The target population for this cross-sectional study was all the 2180 civil servants working in the departments related to the municipality of a city in Toyama Prefecture, Japan, in the spring of 2001. The response rate for questionnaire survey was 80.7% (1759 subjects, of whom 936 (53.2%) were men and 823(46.8%) were women). We also collected urine samples upon awakening in the morning for VMA measurement. The response rate for VMA measurement was 74.9% (1634 subjects). The overall response rate for those subjects with data on both questionnaire and urinary VMA level reached to 70.8% (1544 subjects, of whom 823 (53.3%) were men and 721 (46.7%) were women).

This follow up study is in collaboration with the Whitehall II study,^20^ which was set up to investigate the degree and causes of the social gradient in morbidity and mortality in a cohort of civil servants in London.

Civil servants were classified into four groups based on the job classification system used in the census of the national survey:^21^ 1) administrative; 2) professional; 3) clerical; and 4) protective, transportation and telecommunication service workers which we put them under the category of office support. We also stratified the age of the participants into four groups: <29, 30-39, 40-49 and 50+ years of age. Usual working and sleeping hours per day during the month prior to the study plus the smoking habit was asked through a self-reported questionnaire.

### Job strain

The questionnaire used by Bosma^7^ to report the work environment characteristics (based on Karasek’s job strain model) in the Whitehall II study was adopted for this study to assess job strain (see appendix). Face and content validity plus internal consistency-reliability (see appendix) was satisfactory for the scale, which had already been used in other fields in Japan and also in other countries. We created the four job strain groups constructing a high and low group for demand and control dimensions by dividing the distributions of these dimensions at their respective medians and cross-classifying subjects. Figure shows the resultant four job strain groups.

**Figure.**
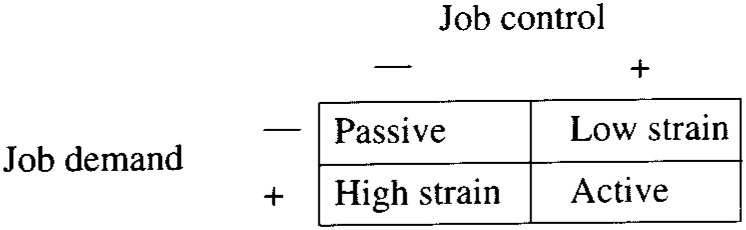
Job-strain model.

### Vanilmandelic acid

The first morning urine specimen (5-6ml) upon awakening was collected by the subject themselves at their home. Each collection tube contained 0.8ml of 6N hydrochloric acid to acidify the urine to a pH less than 1; the urine samples were collected upon arrival of the subjects at the annual health check-up place and kept in iceboxes before being refrigerated at -80°C until analysis. VMA was analyzed with HPLC by modified method of Pisano^22^ with Kawaguchi’s^23^ analyzer at BML company in Saitama, Japan. The urine samples of 40 subjects were divided and analyzed in separate days to assess reproducibility of the measurement. The function of the assay was found to be satisfactory as its intraassay coefficient of variation was 1.11 and the mean difference between the measurements was 0.03 (95% confidence interval -0.01-0.13). The VMA level was adjusted for the creatinine concentration in the urine and expressed as mg/g.creatinine (mg/g.cre).

### Statistical analysis

We performed univariate analysis using a t-test and a one-way analysis of variance (ANOVA) to compare the mean values of VMA for socio-demographic characteristics and smoking status of the subjects. In addition, a three-way ANOVA was used to detect the differences in VMA among the four groups of the job strain model, adjusting for age and smoking status in both sexes. To test whether there is a multivariate association between working and sleeping hours plus job control and demand with VMA, we conducted a multiple linear regression analysis with VMA as the dependant variable. A two-tailed p value of less than 0.05 was set as the significance level. Data were analyzed using SPSS.

## RESULTS

Table 1 shows the mean values of VMA for socio-demographic characteristics and smoking status in men and women. There was an age dependent distribution of urinary VMA levels in the study population. The mean VMA levels increased with age in females and in males over 50 years of age. Females had higher VMA levels than males in all age groups. There was no significant difference in the urinary VMA levels among various employment and job strain groups. Smokers had significantly lower VMA levels than nonsmokers in both sexes.

**Table 1.** Mean±SD of Vanilmandelic acid (mg/g.cre) for socio-demographic characteristics and smoking status of civil servants.

	Male			Female		
	
	n(%)	Mean±SD	F	n (%)	Mean±SD	F
All subjects	859(52.6)	1.83±0.66***		775(47.4)	2.99±0.67***	
Age (year)						
<29	40(4.7)	1.84±0.42	0.023	96(12.5)	2.69±0.47	<0.001
30∼39	63(7.3)	1.78±0.47		42(5.4)	2.75±0.46	
40∼49	373(43.5)	1.76±0.62		342(44.4)	2.82±0.63	
≧50	381(44.4)	1.91±0.74		290(37.6)	3.31±0.67	
Smoking						
Smoker	445(52.2)	1.74±0.67***		37(4.8)	2.76±0.76*	
Nonsmoker	408(47.8)	1.92±0.64***		729(95.2)	3.00±0.66*	
Employment						
Administrative	104(13.8)	1.86±0.71	0.329	15(2.2)	3.28±0.93	0.217
Professional	223(29.5)	1.87±0.63		166(24.6)	2.99±0.76	
Clerical	177(23.4)	1.77±0.63		393(58.3)	2.95±0.61	
Office support	251(33.2)	1.78±0.66		100(14.8)	3.04±0.65	
Job strain						
Passive	332(40.2)	1.81±0.64	0.942	205(28.3)	2.91±0.71	0.1
Low strain	259(31.4)	1.83±0.70		197(27.2)	3.00±0.66	
High strain	108(13.1)	1.83±0.65		155(21.4)	2.96±0.68	
Active	126(15.3)	1.85±0.62		168(23.2)	3.08±0.61	

* p value for t test <0.05.

*** p value for t test <0.001.

Mean values of VMA did not show any significant difference among the four groups of the job strain model after adjusting for age and smoking habits of the civil servants in both men and women (Table 2).

**Table 2.** Mean±SE of Vanilmandelic acid (mg/g.cre) in the four groups of the job strain model, adjusted for age and smoking status.

	Male			Female		
	
	n(%)	Mean±SE	F	n(%)	Mean±SE	F
Passive	331(40.2)	1.84±0.06	0.851	205(28.4)	2.85±0.08	0.610
Low strain	258(31.3)	1.84±0.07		197(27.3)	2.82±0.07	
High strain	108(13.1)	1.88±0.09		153(21.2)	2.93±0.06	
Active	126(15.3)	1.75±0.11		166(23.1)	2.93±0.08	

Results of the multiple regression analysis (Table 3) show that among the independent variables only age has a significant association with the urinary VMA levels upon awakening in the morning (standardized *β* coefficient=0.087, p<0.05 for men; standardized *β* coefficient=0.338, p<0.001 for women). Other independent variables including sleeping hours, working hours, job demand and control did not associate with the urinary levels of VMA. Partial correlation coefficients between VMA and demand/control ratio were -0.01 and 0.04 for men and women respectively, after adjusting for age.

**Table 3.** Multiple linear regression analysis with Vanilmandelic acid (mg/g.cre) as the dependent variable.

	Male		Female	

	*β* Coefficient	p	*β* Coefficient	p
Age	0.087	0.018	0.338	<0.001
Working hours	-0.029	0.461	0.014	0.809
Sleeping hours	-0.066	0.068	-0.009	0.776
Job demand	0.012	0.764	0.039	0.320
Job Control	0.020	0.591	-0.003	0.949

Meanwhile, VMA levels for current smokers (mean±SE: 1.75±0.06) were significantly lower than nonsmokers’ (mean±SE: 1.93±0.05) after adjusting for age in male civil servants (Table 4). The same trend was found in females (current smokers: 2.65±0.17; nonsmokers; 2.91±0.03) but was not significant due to the small number of female smokers. Mean age of the current smokers for initiation of smoking was 20.7 years. However, there was no significant association between job strain levels and the smoking status of the civil servants. Other life style habits were not related to the urinary levels of VMA.

**Table 4.** Mean±SE of urinary Vanilmandelic acid (mg/g.cre) in current smokers and nonsmokers, adjusted for age.

	Male		Female	

Age (year)	Smoker	Nonsmoker	Smoker	Nonsmoker
<29	1.83±0.19	1.88±0.12	2.70±0.2	2.70±0.07
	(12)	(28)	(10)	(86)
30∼39	1.69±0.12	1.85±0.11	2.46	2.76±0.10
	(28)	(35)	(1)	(41)
40∼49	1.69±0.05	1.84±0.05	2.48±0.21	2.83±0.03
	(193)	(178)	(9)	(330)
≧50	1.80±0.04	2.04±0.05	2.96±0.15	3.33±0.04
	(211)	(166)	(17)	(267)
Total	1.75±0.06*	1.91±0.04*	2.65±0.17 †	2.90±0.03 †
	(444)	(407)	(37)	(724)

Number of subjects in parentheses.

* p<0.05 after adjusting for age in comparison with smokers in male civil servants.

† P=0.15 after adjusting for age in comparison with smokers in female civil servants.

## DISCUSSION

Animal models revealed that chronic psychosocial stress could lead, probably via a mechanism involving excessive sympathetic nervous system activation, to exacerbate atherosclerosis.^4^ There is also evidence that overreactivity of the sympathetic nervous system plays a role in the development of hypertension, particularly in its early stages.^24^ Epidemiologic investigations of variations in heart rate have revealed a relation between increased sympathetic tone and long commuting time or extensive overtime.^25^ Park et al.^26^ found a slight positive association between working hours and low frequency power of heart rate variability and urinary excretion of adrenaline in a study of 238 male engineers in Korea. In another study conducted by Sasaki et al.^27^ on 147 subjects, they reported that the group with longer working hours (68 hours/ week) had lower urinary noradrenaline level for one age group (30-39 years) than the group with shorter working hours. In both studies, however, the measurements were conducted through the usual working day with the knowledge that different levels of acute stress in the hours prior to assessment could divert the results to either direction.

Acute impact of psychosocial stress on increasing sympathoadrenal activity has been addressed more frequently in the literature^28^^-^^30^ due to the ability for controlling research condition.

In our study on a large number of civil servants we found no relationship between morning urinary level of VMA upon awakening and the level of job strain. In addition, working hours in a month prior to measurement did not show a significant association with VMA level. It should be mentioned that although excretion of VMA is less changed by the quantity of diet taken in a day prior to assessment, we could not control the results for the sleeping hours prior to urinary collection (since shorter sleeping hours may exert sympathetic predominance^31^) as our questionnaire was based on the actual sleeping hours in the last month prior to the study. However, the large size of our study population could compensate the impact of those who changed their usual sleeping hours in the night prior to urine collection.

Although job strain did not relate to the morning excretion of VMA, this does not rule out the relationship between chronic stress at work and sympathetic overreactivity. Recently research has begun to focus on other forms of work-related stress. For example, one model views work stress as the outcome of high effort and low reward^32^ which has been correlated with progression of carotid atherosclerosis.^33^ Furthermore acknowledging the importance of work place physical conditions, out of work stress, and personality traits such as anger and hostility, it will be premature to rule out the sympathetic overreactivity to the psychosocial stress without considering the above mentioned variables. Comparing our results with those reports which found significant positive relation between acute psychosocial stress and catecholamine levels,^28^^-^^30^ we hypothesize that the link between job strain and sympathoadrenal overreactivity is transitional rather than constitutional in nature. In this view high job strain is a marker of factors that influence sympathoadrenal overreactivity. The transitional view holds that high job strain persons interact with the environment around them in a way that creates enough stimulation for the sympathetic system to overreact.

Forearm venous plasma noradrenaline concentration increases with age.^34^ Our results also provided almost the same pattern with an increase of VMA levels with age in both men and women. However, previous studies had shown that the increase in plasma noradrenaline levels with aging was confined to long-term cigarette smokers,^35^^-^^37^ whereas values in elderly nonsmokers or young smokers were not different from values obtained in young non-smokers. As for short-term effects, it has been speculated that the sympathetic activation induced by smoking depends on an increased release and/or a reduced clearance of catecholamines at the neuroeffector junctions.^38^ However, the mechanism by which plasma noradrenaline levels increased in long-term smokers is unknown. Accumulating clinical evidence has demonstrated that smoking leads to potent inhibition of both types (A and B) of monoamine oxidase,^39^ an enzyme which metabolizes catecholamines, this evidence can also explain the lower VMA level in smokers.

Veral^40^ reported that platelet monoamine oxidase manifested a significant increase in females compared to males, which may be the cause for the significant difference in the urinary VMA levels between men and women in all age groups of our study population.

In conclusion, despite the limitations in our cross-sectional study and single measurement of only one sympathetic activity marker, we showed that there was not an association between job strain and urinary VMA levels upon awakening in the morning in Japanese civil servants.

## APPENDIX

Two characteristics of the work environment (job control and job demand) were assessed by means of 19 items. Response categories ranged from 1 (often) to 4 (never).

Job control: Nine of the 15 items for job control covered decision authority and six items covered skill discretion. The nine items for decision authority were: Do you have a choice in deciding how you do your job? Do you have a choice in deciding what you do at work? Others take decisions concerning my work; I have a good deal of say in decisions about work; I have a say in my own work speed; my working time can be flexible; I can decide when to take a break; I have a say in choosing with whom I work; I have a great deal of say in planning my work environment. The six items for skill discretion were: Do you have to do the same thing over and over again? Does your job provide you with a variety of interesting things? Is your job boring? Do you have the possibility of learning new things through your work? Does your work demand a high level of skill or experience? Does your job require you to take initiative?

Job demands: With four items: Do you have to work very fast? Do you have to work very intensively? Do you have enough time to do everything? Do different groups at work demand things from you that you think are hard to combine?

Cronbach’s alpha for the two components of Karasek’s job strain model was: 0.77 for job control and 0.68 for job demand.
